# Comparative Evaluation of PCR-Based, LAMP and RPA-CRISPR/Cas12a Assays for the Rapid Detection of *Diaporthe aspalathi*

**DOI:** 10.3390/ijms25115773

**Published:** 2024-05-26

**Authors:** Jiali Dong, Wanzhen Feng, Mingze Lin, Shuzhe Chen, Xiaozhen Liu, Xiaodan Wang, Qinghe Chen

**Affiliations:** 1 School of Breeding and Multiplication, School of Tropical Agriculture and Forestry, Hainan University, Sanya 572025, China; djiali@alu.cau.edu.cn (J.D.); fwz@hainanu.edu.cn (W.F.); linmingze@hainanu.edu.cn (M.L.); chenshuzhe@hainanu.edu.cn (S.C.); xiaozliu@hainanu.edu.cn (X.L.); 2 Sanya Institute of China Agricultural University, Sanya 572025, China; xdwang@cau.edu.cn; 3 Key Laboratory of Green Prevention and Control of Tropical Plant Diseases and Pests, Ministry of Education, Hainan University, Haikou 570228, China

**Keywords:** *Diaporthe aspalathi*, recombinase polymerase amplification (RPA), loop-mediated isothermal amplification (LAMP), CRISPR/Cas12a, visual detection

## Abstract

Southern stem canker (SSC) of soybean, attributable to the fungal pathogen *Diaporthe aspalathi*, results in considerable losses of soybean in the field and has damaged production in several of the main soybean-producing countries worldwide. Early and precise identification of the causal pathogen is imperative for effective disease management. In this study, we performed an RPA-CRISPR/Cas12a, as well as LAMP, PCR and real-time PCR assays to verify and compare their sensitivity, specificity and simplicity and the practicality of the reactions. We screened crRNAs targeting a specific single-copy gene, and optimized the reagent concentrations, incubation temperatures and times for the conventional PCR, real-time PCR, LAMP, RPA and Cas12a cleavage stages for the detection of *D. aspalathi*. In comparison with the PCR-based assays, two thermostatic detection technologies, LAMP and RPA-CRISPR/Cas12a, led to higher specificity and sensitivity. The sensitivity of the LAMP assay could reach 0.01 ng μL^−1^ genomic DNA, and was 10 times more sensitive than real-time PCR (0.1 ng μL^−1^) and 100 times more sensitive than conventional PCR assay (1.0 ng μL^−1^); the reaction was completed within 1 h. The sensitivity of the RPA-CRISPR/Cas12a assay reached 0.1 ng μL^−1^ genomic DNA, and was 10 times more sensitive than conventional PCR (1.0 ng μL^−1^), with a 30 min reaction time. Furthermore, the feasibility of the two thermostatic methods was validated using infected soybean leaf and seeding samples. The rapid, visual one-pot detection assay developed could be operated by non-expert personnel without specialized equipment. This study provides a valuable diagnostic platform for the on-site detection of SSC or for use in resource-limited areas.

## 1. Introduction

Soybean (*Glycine max*) is one of the major global sources of vegetable protein and oil. However, soybean production is threatened by several diseases, resulting in reduced crop yield and quality [1,2]. Among the most important soybean diseases, southern stem canker (SSC), causal pathogen *Diaporthe aspalathi* (formerly known as *Diaporthe phaseolorum* var. *meridionalis*), can significantly impact the yield and quality of this crop [3,4,5]. In severe cases, field losses caused by SSC have reached up to 80% [5]. To effectively manage SSC, early detection technologies are critical for disease diagnosis. This strategic approach aims to prevent the dissemination of the pathogen into disease-free soybean-growing regions [3,6].

The traditional detection method for *D. aspalathi* relies on culture isolation and morphological identification, which is laborious and time-consuming despite providing definitive evidence of infection [6,7]. To overcome the limitations of traditional morphological identification, various molecular technologies including conventional PCR and qPCR have been utilized for pathogen detection [8,9,10]. However, their dependence on well-equipped laboratories and skilled personnel impedes their use for on-site detection and resource-limited environments. Conversely, various isothermal amplification techniques, such as Loop-Mediated Isothermal Amplification (LAMP) and Recombinase Polymerase Amplification (RPA), have overcome the limitations of PCR-based assays [6,11,12]. These methods enable nucleic acid amplification at a constant temperature, thereby supporting field applications for easy-to-use, on-site detection in resource-limited settings [11,13]. However, their application presents several challenges, primarily chiefly related to the possibility of false-positive results caused by unintended aerosol contamination or nonspecific amplification [14,15].

Recently, the Clustered Regularly Interspaced Short Palindromic Repeat (CRISPR) and CRISPR-associated (Cas) system have been found to be significantly applicable in molecular detection [16,17,18,19,20]. Several CRISPR/Cas-based detection platforms, including DETECTR [16], HOLMES [19], and SHERLOCK [18,21], have been developed as point-of-care diagnostic tools enabling higher specificity and sensitivity in pathogen detection. Henceforth, CRISPR/Cas-assisted nucleic acid amplification has demonstrated substantial promising advancements in next-generation molecular detection technologies [22]. However, these platforms separate the nucleic acid amplification and Cas12a cleavage steps, potentially increasing the risk of carryover contamination. To address these challenges, recent efforts have focused on integrating isothermal amplification methods with CRISPR/Cas reactions in single-pot assays [14,23]. This approach serves to diminish the likelihood of cross-contamination, thereby concurrently enhancing assay specificity [14,24,25,26,27]. Recent advancements have leveraged CRISPR/Cas12a methodologies for the detection of plant pathogens [28,29,30,31].

The objective of the current study was to compare the efficiencies of different PCR-based assays alongside the thermostatic detection technologies LAMP and RPA, to determine the most suitable detection system for the on-site diagnosis of *D. aspalathi*. A new target gene (LJJS01001645.1) was identified from the whole-genome sequence of *D. aspalathi* based on public genomic sequence data and bioinformatic analysis. This enabled the development of PCR-based, LAMP and RPA assays for the highly specific detection of *D. aspalathi*. The specificity and sensitivity of the LAMP and RPA-CRISPR/Cas12a assays were assessed in comparison to conventional PCR, qPCR and each other. Finally, the practicality of the LAMP and RPA-CRISPR/Cas12a assays was evaluated using inoculated soybean samples.

## 2. Results

### 2.1. Optimization of LAMP and RPA-CRISPR/Cas12a Assays

**Figure 1:**
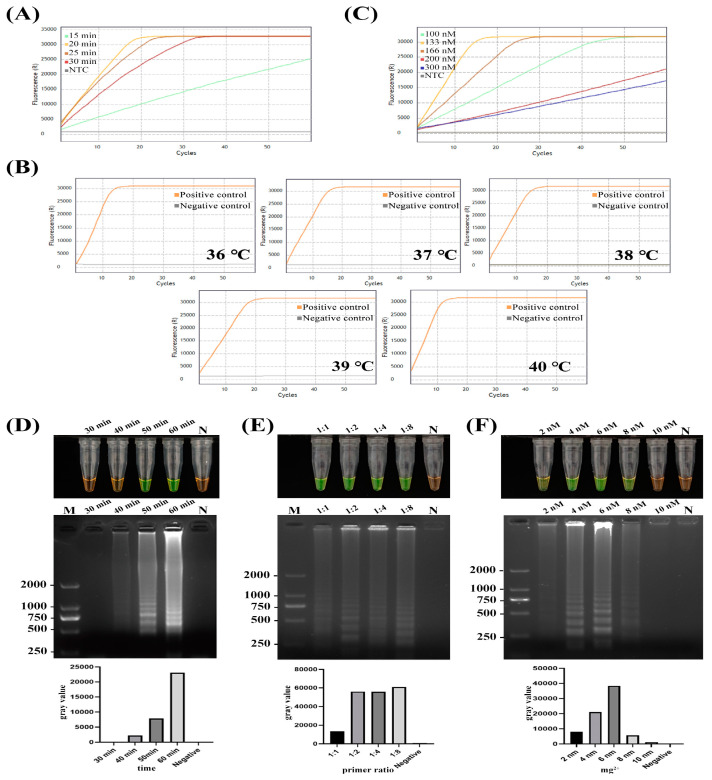
Optimization of RPA-CRISPR/Cas12a and LAMP assays. RPA-CRISPR/Cas12a results under different reaction conditions according to (**A**) different concentrations of Cas12a and sgRNA in the case of Cas12a:sgRNA = 1:1; (**B**) different temperatures of RPA; (**C**) different times of RPA reaction. LAMP results under different reaction conditions according to (**D**) different reaction times of LAMP; (**E**) different ratios of inner and outer primers; (**F**) different concentrations of Mg^2+^. M, DL2000 DNA marker. N, negative control.

To ensure the optimal CRISPR/Cas12a reaction, the concentrations of crRNA and the ratio of Cas12a to crRNA were first optimized in the CRISPR/Cas12a assay, as crRNA concentration is crucial for the CRISPR/Cas12a reaction. The optimal crRNA concentration and Cas12a to crRNAs ratio were determined to be 133 nM and 1:1, respectively (Figure 1A). Using these optimized crRNA and Cas12a conditions, the RPA reaction time and temperature were further optimized. Results demonstrated that the optimal temperature range for the RPA reaction was 36~40 °C (Figure 1B). Consequently, 37 °C was selected for subsequent experiments, aligning with the ideal temperature for Cas12a enzyme activity. The ideal reaction time was determined to be 20 min (Figure 1C). Major parameters were also examined to optimize reaction conditions for the LAMP assay. The LAMP assay was performed with varying reagent concentrations, inner to outer primer ratios, and reaction times to determine the optimal system. For *D. aspalathi* target DNA, the optimal reaction temperature and time were 65 °C and 60 min, respectively (Figure 1D). The optimal primer ratio (inner to outer) was determined to be 1:8 (Figure 1E), and the ideal Mg^2+^ concentration was 6 nM (Figure 1F). Amplified products were sequenced for confirmation, with the obtained sequences perfectly matching the expected DNA sequence of the LJJS01001645.1 target gene. In this study, the RPA reaction results were not significantly influenced by temperatures ranging from 36 to 40 °C. However, an optimized temperature of 37 °C was selected, as it was suitable for both the RPA and Cas12a/crRNA reactions. Utilizing this standardized optimal temperature facilitated precise control over reaction conditions and facilitated the feasibility of the one-pot detection assay. Operating under a constant temperature regimen further enhanced the adaptability of the assay for on-site detection purposes.

### 2.2. Establishment of One-Pot RPA-CRISPR/Cas12a Assay

**Figure 2:**
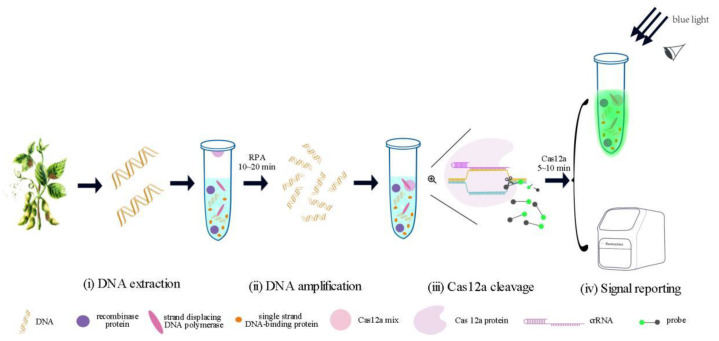
Schematic diagram of the RPA-CRISPR/Cas12a assay workflow for rapid, visual one-pot detection of *D. aspalathi*. Cas12a-based detection involves (**i**) extraction of DNA from SSC disease samples; (**ii**) RPA employed to specifically amplify the target gene from DNA, (**iii**) Cas12a nuclease to cleave the amplified target DNA (cis-cleavage), and (**iv**) the generation of fluorescence that can be observed by the naked eye.

To enable the practical on-site detection of *D. aspalathi*, a unified one-pot RPA-CRISPR/Cas12a assay was developed, integrating RPA amplification and CRISPR/Cas12a cleavage in a single reaction tube, as depicted in Figure 2. The detection platform involves four steps: (i) nucleic acid extraction, (ii) target DNA amplification via RPA, (iii) CRISPR/Cas12a-mediated cleavage by Cas12a/crRNA, and (iv) fluorescent signal output. First, total genomic DNA is extracted from *D. aspalathi*-infected plant samples. The reaction mixtures of RPA and CRISPR/Cas12a are subsequently segregated into the bottom and lid compartments of the reaction tube, respectively. The RPA amplification reaction attained completion in 20 min. Subsequently, the RPA reaction mixture is combined with the CRISPR/Cas12a reagents on the tube lid, allowing amplicons to be recognized by the Cas12a/crRNA system. Finally, Cas12a targeting of the double-stranded DNA (dsDNA) initiates its collateral nuclease activity, resulting in the cleavage of the single-stranded DNA (ssDNA) reporter and the subsequent emission of a fluorescent signal. The result is determined by a visual observation of fluorescence under blue or UV light. Through these steps, a one-pot visual RPA-CRISPR/Cas12a assay for the detection of *D. aspalathi* can be established. A typical workflow of the platform is illustrated in Figure 2.

**Figure 3:**
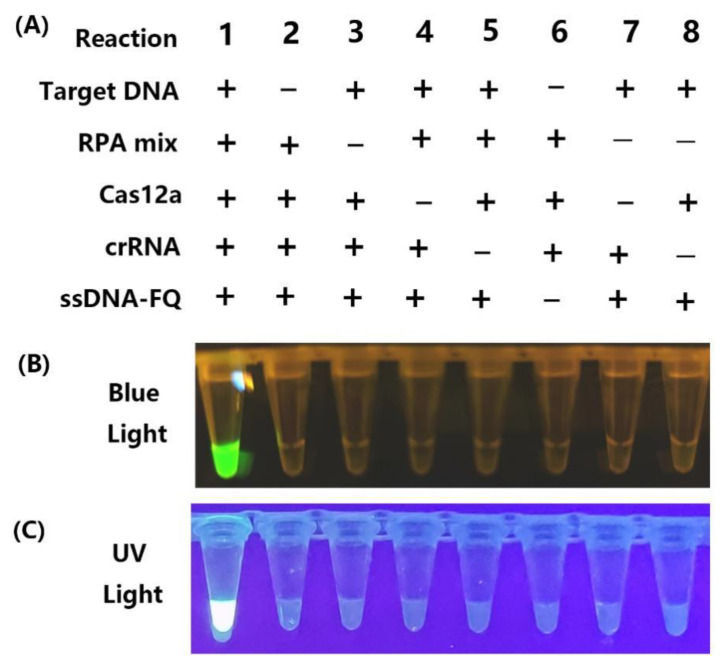
Evaluation of RPA-CRISPR/Cas12a reaction systems with various components. (**A**) Eight RPA-CRISPR/Cas12a reactions with various components; (**B**) visualization under blue light after 30 min incubation; (**C**) visualization under UV light after 30 min incubation. Each experiment was repeated three times with similar results.

To systematically evaluate the one-pot RPA-CRISPR/Cas12a assay, eight distinct reaction systems (referred to as reactions #1–8) containing different components were prepared, and subsequently subjected to testing (Figure 3A). Genomic DNA containing the target sequence was utilized as the template. The ssDNA-FQ reporter was labeled with a 5′ FAM fluorophore and 3′ BHQ-1 FQ quencher. As depicted in Figure 3, after 20 min of RPA amplification followed by 5~10 min of CRISPR/Cas detection, only reaction #2, containing target DNA, the RPA reaction mixture, Cas12a, crRNAs, and ssDNA-FQ, exhibited bright fluorescence under blue light (Figure 3B) or UV light (Figure 3C). Therefore, these results demonstrate that the one-pot CRISPR/Cas12a assay provides a rapid, specific, and simple method for detecting *D. aspalathi*. Visual detection holds paramount importance in molecular diagnostics, particularly in resource-limited environments. In this study, a fluorescence reporter molecule, labeled with a 5′ FAM fluorophore and 3′ BHQ1 quencher, was employed to visually assess the CRISPR/Cas12a detection outcomes. This direct observation method facilitates the straightforward and expeditious interpretation of results, with an accuracy level comparable to instrument-based detection.

### 2.3. Specificity of PCR-Based, LAMP and RPA-CRISPR/Cas12a Assays

**Figure 4:**
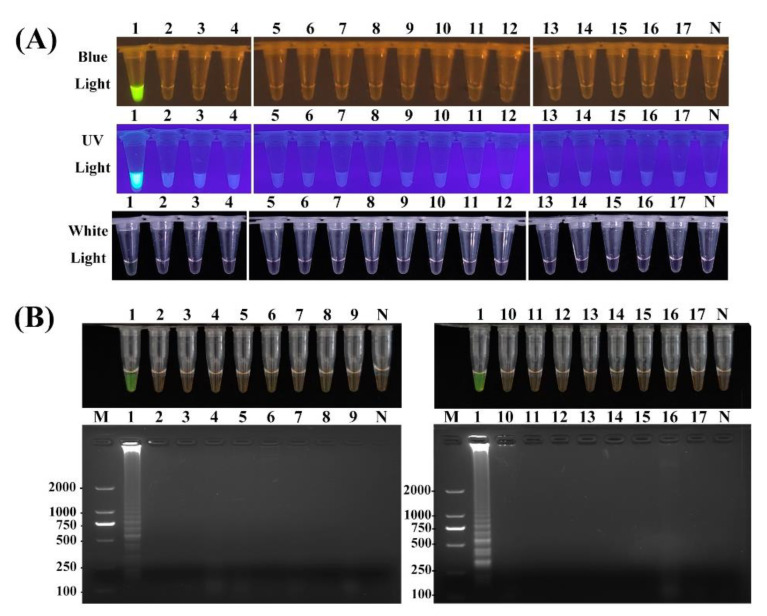
Specificity of RPA-CRISPR/Cas12a and LAMP assay for the detection of *D. aspalathi*. The specificity was confirmed by (**A**) RPA-CRISPR/Cas12a test results under blue light, UV light and white light; (**B**) LAMP test results of color changes and agarose gel electrophoresis. M, DL2000 DNA marker. N, negative control. Lane 1, *Diaporthe aspalathi*; Lane 2, *D. caulivora*; Lane 3, *D. longicolla*; Lane 4, *Phytophthora sojae*; Lane 5, *Pythium aphanidermatum*; Lane 6, *Colletotrichum glycines*; Lane 7, *C. destructivum*; Lane 8, *C. fructicola*; Lane 9, *Rhizoctonia solani*; Lane 10, *Fusarium graminearum*; Lane 11, *F. solani*; Lane 12, *F. oxysporum*; Lane 13, *F. equiseti*; Lane 14, *F. proliferatum*; Lane 15, *F. virguliforme*; Lane 16, *Sclerotinia sclerotirum*; Lane 17, *Alternaria brassicae*.

To assess the specificity of the assays, DNA templates extracted from isolates of the target pathogen *D. aspalathi* and 17 non-target species including close-related fungi were assessed. As depicted in Figure 4 and Figure S1, after PCR, qPCR, LAMP or RPA-CRISPR/Cas12a assay, positive amplification was observed exclusively for the target *D. aspalathi* isolates. No amplification was detected for the non-target fungal species tested, including the closely related species *D. longicolla* and *D. caulivora*, or other soybean-associated fungi (Figure 4A). No cross-reactivity was observed. As expected, these results were consistent from the result of agarose gel electrophoresis analysis (Figure 4B), which revealed amplification products only for the target *D. aspalathi* isolates. Collectively, these results demonstrate that both the optimized LAMP and RPA-CRISPR/Cas12a assays established in this study exhibited high specificity for *D. aspalathi*.

The integration of CRISPR/Cas12a can mitigate non-specific amplification in RPA reactions and improve the overall detection sensitivity. The enhanced reliability and precision of CRISPR-based detection could be attributed to the dual target recognition through both RPA primers and could guide RNA hybridization, which enables the specific discrimination of the target pathogen *D. aspalathi* from other closely related fungal species (Figure 4A). Overall, the coupled target binding of the guide RNA and RPA primers confers exquisite analytical specificity due to the dual hybridization requirement for the activation of the Cas12a trans-cleavage reporter system.

### 2.4. Comparison of Sensitivity

**Figure 5:**
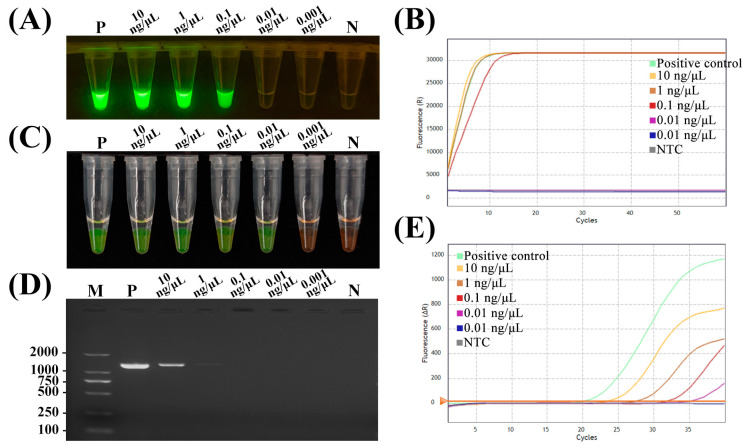
Sensitivity of RPA-CRISPR/Cas12a and LAMP assay for the detection of *D. aspalathi*. The sensitivity was evaluated by (**A**,**B**) RPA-CRISPR/Cas12a; (**C**) LAMP; (**D**) PCR; (**E**) qPCR. M, DL2000 DNA marker; P, positive control; N and NTC, negative control.

To comprehensively evaluate analytical sensitivity, 10-fold serial dilutions of target DNA, ranging from 100 ng μL^−1^ to 0.001 ng μL^−1^, were subjected to testing by RPA-CRISPR/Cas12a, LAMP, conventional PCR and qPCR. As shown in Figure 5 and Table 1, RPA-CRISPR/Cas12a and LAMP assays could detect as low as 0.1 ng μL^−1^ and 0.01 ng μL^−1^ target DNA, respectively. Conventional PCR exhibited a 10-fold lower sensitivity limit of 1.0 ng μL^−1^ target DNA. The qPCR assay displayed a sensitivity matching that of RPA-CRISPR/Cas12a, detecting 0.1 ng μL^−1^ of target DNA. Although LAMP achieved a 10-fold greater analytical sensitivity relative to RPA-CRISPR/Cas12a, the latter assay enabled the coupling of CRISPR/Cas12a-mediated DNA cleavage and amplification in a streamlined single-tube reaction at 37 °C. Despite marginally lower sensitivity, this unique capability of RPA-CRISPR/Cas12a confers substantial advantages for expedient pathogen detection and the promotion of field-based molecular diagnostics. In summary, while LAMP attained the highest analytical sensitivity, the RPA-CRISPR/Cas12a assay performed comparably to the gold-standard qPCR, with the added benefit of having simpler, isothermal single-tube implementation. The sensitivity and simplicity of RPA-CRISPR/Cas12a highlight its potential as a powerful tool for the rapid, accessible molecular detection of plant pathogens.

### 2.5. Validation of RPA-CRISPR/Cas12a and LAMP Detection Using Disease Samples

**Figure 6:**
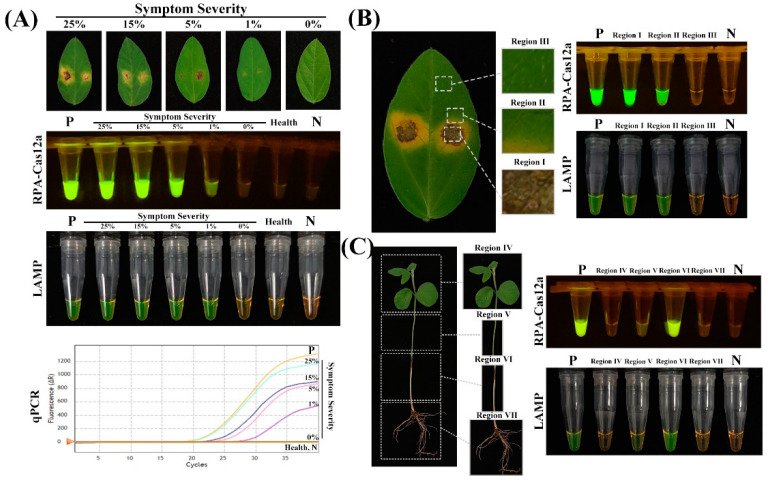
Detection of *D. aspalathi* in infected plant samples using the RPA-CRISPR/Cas12a, LAMP and qPCR. (**A**) Different levels of symptom severity; (**B**) different parts of infected soybean leaf; (**C**) different parts of soybean plant. P, positive control. N, negative control.

To evaluate the practical applicability of the developed RPA-CRISPR/Cas12a and LAMP assays, infected soybean leaf samples exhibiting a range of disease severities following inoculation with *D. aspalathi* were assessed. As shown in Figure 6A, both RPA-CRISPR/Cas12a and LAMP assays could detect the target *D. aspalathi* pathogen in infected detached leaves exhibiting just 1% symptom severity, and the results were consistent with qPCR-based diagnoses. Three distinct leaf regions (I, II, III) were tested by both assays, revealing clear green fluorescence amplification signals in regions I and II, but not in the symptomless region III or the negative control (Figure 6B). Inoculated soybean seedlings were divided into four distinct groups (region IV, V, VI, VII) and tested by both assays. Consistent pathogen detection was achieved in regions V and VI, but not in regions IV and VII, by RPA-CRISPR/Cas12a and LAMP (Figure 6C). The experiments were replicated three times with consistent results. The detection results in both assays were all consistent with the results obtained by the qPCR method (Figure 6A). Collectively, these results confirm the capability of the rapid, visual RPA-CRISPR/Cas12a and LAMP assays to detect the target *D. aspalathi* pathogen directly from infected plant material. While LAMP assays require high-temperature incubation at around 60–65 °C, a key advantage of the RPA-CRISPR/Cas12a approach is the ability to perform rapid target detection at 37 °C, consistent with normal human body temperature. By avoiding cumbersome heating equipment, this isothermal reaction at 37 °C confers significant potential to further reduce dependence on instrumentation and enable large-scale on-site testing in resource-limited settings. Therefore, the RPA-CRISPR/Cas12a assay exhibits unique practical benefits for the decentralized, field-based molecular diagnosis of plant pathogens.

## 3. Discussion

Outbreaks of southern stem canker (SSC) are a constant threat to soybean production worldwide [3,4]. A rapid and efficient field detection method is urgently needed to prevent and reduce the losses. Currently, detection methods for SSC remain confined to low-sensitivity PCR and other complex equipment-dependent detection techniques [8,10]. In order to advance the prediction and prevention strategies for this disease, we have devised two novel, more efficient and convenient detection methodologies.

In this study, a highly specific target gene was identified by comparing the whole-genome sequence of *D. aspalathi* and various other pathogens. A large number of experimental results have shown that the optimized LAMP and RPA-CRISPR/Cas12a assays based on this specific target gene exhibit high specificity compared to conventional PCR and qPCR [15,32,33]. LAMP and RPA-CRISPR/Cas12a operate at a constant temperature with reduced dependency on specialized instruments [12]. RPA is proven to be more convenient and cost-effective than LAMP, as it requires only a single primer pair, in contrast to 4~6 primers required for LAMP. Additionally, RPA offers the advantage of short reaction times compared to LAMP and other isothermal techniques. Furthermore, the RPA amplification temperature aligns seamlessly with the CRISPR/Cas12a nuclease activity (37 °C), allowing for integration into a unified one-pot reaction. Therefore, RPA-CRISPR/Cas12a was favored over LAMP-CRISPR/Cas12a for the development of the one-pot *D. aspalathi* detection assay, owing to the compatibility of RPA amplification and Cas12a detection at the same temperature. However, previous reports have demonstrated that CRISPR/Cas12a-based assays generally exhibit higher specificity and sensitivity compared to RPA-only detection methods [26,34]. In our findings, it was observed that LAMP exhibited greater sensitivity compared to RPA-CRISPR/Cas12a. However, upon analyzing the detection outcomes of the diseased samples, no significant difference in sensitivity between the LAMP and RPA-CRISPR/Cas12a assays was discerned. Although both methods optimized the detection of *D. aspalathi*, compared to LAMP, which requires 65 °C and more than three pairs of primers, RPA-CRISPR/Cas12a only requires one pair of primers and the entire experimental process can be executed under normal-body-temperature conditions. This attribute renders RPA-CRISPR/Cas12a particularly advantageous for field applications.

The two experimental methods established in this article significantly reduce the requirements for experimental instruments and reagents compared to traditional PCR and qPCR [8,11]. The main beauty of our research is that RPA-CRISPR/Cas12a can be achieved in one pot under normal body temperature conditions (37 °C) within 30 min, and can therefore be used to detect SSC without equipment in the field. Therefore, our developed methods have high application potential for SSC disease diagnosis and the prevention of spread into disease-free soybean growing regions. They can also provide experimental and theoretical bases for the detection of other diseases, which is of great significance in field detection.

Despite the results presented here being satisfactory, we expect several technical enhancements in the future for the RPA-CRISPR/Cas12a assay. On the one hand, although the RPA-CRISPR/Cas12a assay developed offers time-saving and simplicity advantages, it currently lacks the capability to concurrently detect multiple targets within a single reaction tube. The aim of introducing a physical device with multiplex channels would be to facilitate the detection of different regions for CRISPR/Cas detection by segregating the multiplex Cas/crRNA targeting multiple targets. On the other hand, all reagents in the RPA-CRISPR/Cas12a assay can be stored at room temperature, eliminating the need for cold chains and allowing for rapid detection in a non-laboratory setting.

Further improvements and advancements will involve integrating our RPA-CRISPR/Cas12a assay into a disposable microfluidics chip platform, thereby enabling fully integrated, sample-to-result and multiplexed detection capabilities. Given the strong fluorescence signals generated by our RPA-CRISPR/Cas12a assay at the endpoint, there exists the possibility of recording, analyzing and reporting detection results utilizing ubiquitous smartphone technology [35,36]. By leveraging smartphones, equipped with the capability to capture fluorescence photos and convert them into fluorescence intensity data, along with analytical algorithms, qualitative or semiquantitative test results can be obtained. Moreover, these results can be wirelessly transmitted to a designated website or remote server, accompanied by GPS coordinates, thereby providing access to experts and farmers. This integrated approach is crucial for facilitating simple, rapid, intelligent and connected plant disease diagnostics.

## 4. Materials and Methods

### 4.1. Materials and Reagents

Soybean seeds (cv. Willimas) were obtained from the plant pathology department, Hainan University. LbaCas12a nuclease and 10× NEBuffer2.1 were acquired from New England Biolabs (Beijing, China). The recombinase polymerase amplification (RPA) basic kit was purchased from Amp-Future Biothech Co., Ltd. (Changzhou, China). Bst DNA Polymerase, ThermoPol^®^ Reaction Buffer Pack, and MgSO_4_ were obtained from New England Biolabs (Beijing, China). In addition, 2 × Taq PCR Master Mix II was purchased from Tiangen Biotech (Beijing, China). High Affinity HotStart Taq, 10×HA Buffer and 5 × Probe qPCR Buffer were also acquired from Tiangen Biotech (Beijing, China). All primers and DNA fragments (including crRNA, and ssDNA probes) were synthesized by Sangon Biotech Co., Ltd. (Shanghai, China). Nuclease-free H_2_O was obtained from Sangon Biotech (Shanghai, China). Genomic DNA extraction kits were procured from Tiangen Biotech (Beijing, China).

### 4.2. Isolates and DNA Extraction

Single-spore isolates of plant pathogens either isolated from soybean or acquired from the School of Tropical Agriculture and Forestry, Hainan University, and the Post-Entry Quarantine Center for Tropical Plant, Haikou Customs District, China, were used in this study. All isolates were routinely cultured on potato-dextrose agar media at a temperature of 20 °C in the dark.

Genomic DNA was extracted from mycelial cultures using the DNA extraction kit (Tiangen Biotech Co., Ltd., Beijing, China), as described previously [8]. The quantity of extracted gDNA was estimated using a NanoDrop™ One Microvolume UV–Vis Spectrophotometer (Thermo Fisher Scientific Inc., Waltham, MA, USA), and aliquots were stored at a concentration of 100 ng μL^−1^ in sterile distilled water at −20 °C

### 4.3. Primer and crRNA Design

To identify conserved, single-copy candidate detection targets specific for *D. aspalathi* assays, all gene sequences of *D. aspalathi* were utilized to perform BLAST searches against publicly available genomic sequences of *Diaporthe* and related taxa. Ultimately a single-copy gene (LJJS01001645.1) was selected as the specific target for the RPA, LAMP, conventional PCR and qPCR assays. Primers of RPA (D-RPA-1F/D-RPA-1R), LAMP (D1-F3/D1-B3/D1-FIP/D1-BIP), PCR (Da-1F/Da-1R) and qPCR (Da-Q3-F1/Da-Q3-R1/Da-Q3) were designed and analyzed by primer 5 and Primer-Explore V5 software (http://primerexplorer.jp/e/) (accessed on 5 March 2022) separately. The crRNA consisted of a 20~24 bp target-dependent sequence following the PAM motif (5′-TTTN-3′) and was designed using EuPaGDT (http://grna.ctegd.uga.edu) (accessed on 10 May 2022). An RNA scaffold assisted binding to the Cas protein. The primer sequences are enumerated in Table 2, providing comprehensive information for their utilization.

### 4.4. Conventional PCR Detection of D. aspalathi

Conventional PCR assays were performed in 20 µL reactions, with each reaction comprising 10 µL of 2 × Taq PCR Master Mix, along with 0.5 µL each of forward and reverse primers (Da-1F/Da-1R) (10 µM), 1 µL of template DNA (100 ng µL^−1^), and 8.0 µL of nuclease-free H_2_O. Thermal cycling was conducted using a PTC-200 Thermo Cycler (MJ Research, Watertown, MA, USA) under the following conditions: an initial denaturation at 94 °C for 5 min, followed by 30 cycles of denaturation at 94 °C for 30 s, annealing at 56 °C for 30 s and extension at 72 °C for 30 s, with a final extension at 72 °C for 10 min.

### 4.5. Real-Time PCR Detection of D. aspalathi

Real-time PCR assays were performed in 20 µL reactions. Each reaction was composed of 2 µL of 10 × HABuffer, 4 µL of 5 × probe qPCR Buffer, 1.6 µL of dNTPs (2.5 mM each), 0.2 µL of High Affinity HotStart Taq, 0.5 µL each of forward and reverse primers Da-Q3-F1/Da-Q3-R1 (10 µM), 0.5 µL of Da-Q3 probe (10 µM), 1 µL of template DNA (100 ng µL^−1^) and 9.7 µL of RNase-free H2O. Thermal cycling was carried out on an AriaMx qPCR System (Agilent Technologies, Inc., Santa, CA, USA) under the following conditions: initial denaturation at 95 °C for 3 min, followed by 40 cycles of denaturation at 95 °C for 5 s and annealing at 60 °C for 30 s.

### 4.6. Optimization of LAMP and RPA-CRISPR/Cas12a Assays

The efficiency of the CRISPR/Cas12a cleavage system is directly influenced by the concentrations of crRNA and Cas12a, impacting both trans-cleavage efficiency and fluorescence intensity. To ascertain the optimal conditions for the RPA-CRISPR/Cas12a assay, a range of Cas12a (100~300 nM) and crRNA (100~300 nM) concentrations, RPA reaction temperatures (36~40 °C) and RPA reaction times (15~30 min) were evaluated. For the optimization of the LAMP assay, four different reaction periods (30~60 min), four ratios of inner and outer primers (F3/B3:PFIP/BIP = 1:1; 1:2; 1:4; 1:8) and varying Mg^2+^ concentrations (2~10 nM) were tested. Nuclease-free H_2_O was employed as a negative control. The LAMP and CRISPR/Cas12a reaction products were analyzed through naked-eye observation or by detecting the maximal fluorescence signal value to determine the optimal reaction conditions. All experiments were performed in triplicate.

### 4.7. LAMP Assay

The LAMP reaction was performed in 25 μL total volume containing the following: 2.5 μL 10 × ThermoPol Buffer, 2 μL 100 mM MgSO_4_, 1 μL Bst DNA polymerase, 3.5 μL each of 10 mM dNTPs, 2 μL 10 mM betaine, 0.5 μL each of 10 nM primers, 1 μL of template DNA (100 ng μL^−1^) and 10 μL of nuclease-free H_2_O. Negative controls containing nuclease-free H_2_O instead of template DNA were included in each assay. After the addition of all reagents, reaction tubes were incubated at 65 °C for 60 min. All experiments were performed in triplicate.

### 4.8. RPA-CRISPR/Cas12a Assay

The RPA-CRISPR/Cas12a assay integrated RPA amplification with Cas12a digestion within a unified one-pot reaction system. The RPA reaction was conducted according to the kit instructions provided (WLB8201KIT, AMP-Future Biotech Co., Ltd., Weifang, China). Briefly, each 10 µL RPA reaction contained 5.9 µL of buffer A, 0.5 μL buffer B, 1 μL template DNA (100 ng µL^−1^), 0.4 µL each of forward and reverse primers (10 µM) and 1.8 μL nuclease free H_2_O. The 5 μL CRISPR/Cas12a reaction solution contained 1.0 μL crRNA (2 μM), 1.0 μL Cas12a (2 μM), 1.0 μL ssDNA FQ reporter (100 nM), 1.5 μL 10 × NEB Buffer 2.1 and 0.5 μL of nuclease-free H2O. The Cas12a mixture was initially placed inside the lid of the tube. After the RPA reaction at 37 °C for 20 min, the Cas12a solution was centrifuged into the RPA solution, followed by an additional incubation of 5~10 min at 37 °C. Positive reactions will exhibit green fluorescence signals under blue or UV light.

### 4.9. Specificity Test

To examine the specificity of the RPA-CRISPR/Cas12a and LAMP methods, DNA templates extracted from *D. aspalathi* isolates and 16 non-*D. aspalathi* isolates, including closely related species *D. longicolla* and *D. caulivora*, were used to evaluate specificity. The nuclease-free H_2_O served as the negative control. All experiments were conducted in triplicate.

### 4.10. Comparison of Sensitivity

To further assess the sensitivity of both the RPA-CRISPR/Cas12a and LAMP assays, gDNA was serially diluted in 10-fold dilutions to generate five concentrations (10.0, 1.0, 0.1, 0.01 and 0.001 ng μL^−1^) in sterile distilled water. To compare the sensitivity of the two assays to other nucleic acid detection methods, PCR and qPCR were tested using the same DNA concentrations. Nuclease-free H_2_O served as a negative control. All assays were conducted in triplicate for each gDNA concentration.

### 4.11. Feasibility of LAMP and RPA-CRISPR/Cas12a Detection Using Disease Samples

To verify the validity and feasibility of LAMP and RPA-CRISPR/Cas12a detection for *D. aspalathi*, soybean seedlings were inoculated with *D. aspalathi*, as described previously [8]. An equivalent number of plants, treated with sterile agar plugs, were employed as negative controls. Soybean-detached leaves were inoculated and divided into groups exhibiting different symptom severities (0, 1, 5, 15 and 25%), with each leaflet divided into three regions from the inoculation site to the leaf apex. Soybean seedlings were inoculated and divided into four regions for analysis: root, lower stem, upper stem and leaf. DNA was extracted from each sample and evaluated using the LAMP and RPA-CRISPR/Cas12a assays. Each experiment was performed in triplicate.

## 5. Conclusions

In our comparison of PCR, qPCR and RPA-Cas12a assays, LAMP showed the greatest sensitivity, followed by qPCR and RPA-Cas12a. The two thermostatic methods, RPA-Cas12a and LAMP assays, were validated to detect the soybean pathogenic fungus *D. aspalathi* using infected soybean leaf and seeding samples. The streamlined one-tube format helps to preclude contamination while providing straightforward visual readout. The assays exhibit analytical performance comparable to quantitative PCR, with the advantages of simpler implementation under field conditions. Overall, this study establishes a robust molecular diagnostic platform with strong potential for SSC diagnosis in resource-limited settings.

## Figures and Tables

**Table 1:** Comparison of conventional PCR, qPCR, LAMP and RPA-CRISPR/Cas12a sensitivity in this study.

	1.00 × 10^2^ ng μL^−1^	1.00 × 10^1^ ng μL^−1^	1.00 × 10^0^ ng μL^−1^	1.00 × 10^−1^ ng μL^−1^	1.00 × 10^−2^ ng μL^−1^	1.00 × 10^−3^ ng μL^−1^
Conventional PCR	+	+	+	−	−	−
qPCR	+	+	+	+	−	−
LAMP	+	+	+	+	+	−
RPA-Cas12a	+	+	+	+	−	−

**Table 2:** Sequences of the primers and a probe used in this study.

Primer	Sequence	Reference
Da-1F	GAATCCTTGTGGGTATTTG	For PCR
Da-1R	GTCAATATGCTATGGTCAC	
Da-Q3-F1	GCGATTTGTGGGATTGAC	For qPCR
Da-Q3-R1	CATGCTGAATAGGAGAGG	
Da-Q3	ROX-CCGTCAAGGCTACACTCGTCG-BHQ2	
D1-F3	GTGCCTCGAACGTTGTCTC	For LAMP
D1-B3	GGCGACACAAACACGGAC	
D1-FIP	ACCCGAGGGGGAAGTTCAAACTTCGTAGTTCTGCCAAGAAGC	
D1-BIP	CTGCGGTCCCTGGAGAGGATACTTCATGTAGCGCCCGA	
D-RPA-1F	CTTTGTCTGAGGCTGAACCCCAGGCGTATT	For RPA
D-PRA-1R	CGCATCAACAACCAAGATCACGGGCAAGAC	
D-Cas-C	UAAUUUCUACUAAGUGUAGAUCCGGAUGCGUACAACAUGACA	For Cas12a cleavage

## Data Availability

The original contributions presented in the study are included in the article/supplementary materials, further inquiries can be directed to the corresponding author/s.

## Supplementary Materials

The supporting information can be downloaded at: https://www.mdpi.com/article/10.3390/ijms25115773/s1.
