# Supplementary material for: Comparative Evaluation of PCR-Based, LAMP and RPA-CRISPR/Cas12a Assays for the Rapid Detection of Diaporthe aspalathi
Source: Int J Mol Sci. 2024 May 26;25(11):5773. doi: 10.3390/ijms25115773 (PMC11172161; doi:10.3390/ijms25115773)
Supplement: Supplementary file 1 [file ijms-25-05773-s001.zip › ijms-2983469-supplementary.pdf]

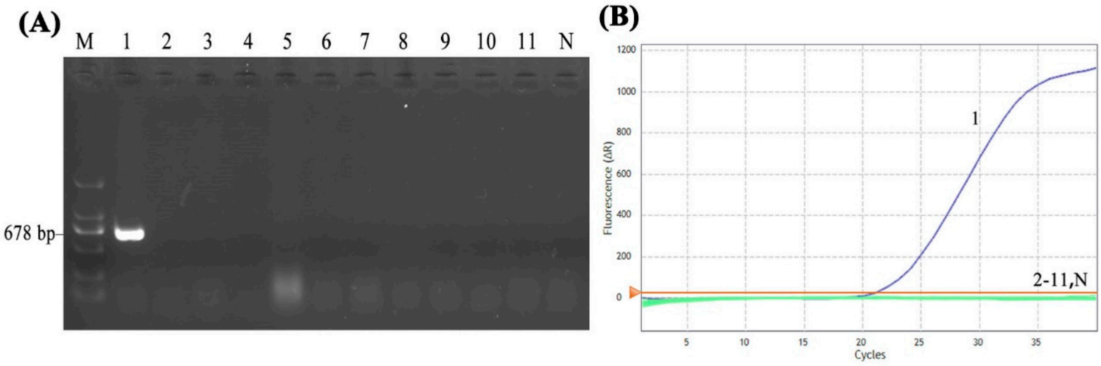

Figure S1. Specificity of PCR and qPCR assay for detection of *D. aspalathi*. The specificity was confirmed by (A) PCR; (B) qPCR. M, DL2000 DNA marker. N, negative control. Lane 1, *Diaporthe aspalathi*; Lane 2, *D. caulivora*; Lane 3, *Fusarium virguliforme*; Lane 4, *Phytophthora sojae*; Lane 5, *Pythium aphanidermatum*; Lane 6, *Colletotrichum glycines*; Lane 7, *Colletotrichum destructivum*; Lane 8, *Colletotrichum fructicola*; Lane 9, *Rhizoctonia solani*; Lane 10, *Fusarium graminearum*; Lane 11, *Fusarium oxysporum.*
